# Supplementary material for: High-frequency repetitive transcranial magnetic stimulation at dorsolateral prefrontal cortex for migraine prevention: A protocol for a systematic review of controlled trials
Source: PLoS One. 2021 Jun 17;16(6):e0251528. doi: 10.1371/journal.pone.0251528 (PMC8211237; doi:10.1371/journal.pone.0251528)
Supplement: S1 Checklist — (DOC) [file pone.0251528.s001.doc]

**PRISMA-P (Preferred Reporting Items for Systematic review and Meta-Analysis Protocols) 2015 checklist: recommended items to address in a systematic review protocol***

| Section and topic | Item No | Checklist item |
| --- | --- | --- |
| ADMINISTRATIVE INFORMATION | | |
| Title: |  |  |
| Identification | 1a | Identify the report as a protocol of a systematic review |
| Update | 1b | If the protocol is for an update of a previous systematic review, identify as such  High-frequency repetitive transcranial magnetic stimulation at dorsolateral prefrontal cortex for migraine prevention: A protocol for a systematic review of controlled trials |
| Registration | 2 | If registered, provide the name of the registry (such as PROSPERO) and registration number  The International Prospective Register of Systematic Reviews (PROSPERO) registration number for this systematic review is CRD42020220636. |
| Authors: |  |  |
| Contact | 3a | Provide name, institutional affiliation, e-mail address of all protocol authors; provide physical mailing address of corresponding author   | No. | Author | E-mail address | Institutional affiliation | | --- | --- | --- | --- | | 1. | Nabil Izzaatie Mohamad Safiai | nabil.izzaatie@student.upm.edu.my | Universiti Putra Malaysia | | 2. | Nur Afiqah Mohamad | m.nurafiqah@upm.edu.my | Universiti Putra Malaysia | | 3. | Hamidon Basri | hamidon@upm.edu.my | Universiti Putra Malaysia | | 4. | Liyana Najwa Inche Mat | liyananajwa@upm.edu.my | Universiti Putra Malaysia | | 5. | Fan Kee Hoo | fan_kee@upm.edu.my | Universiti Putra Malaysia | | 6. | Anna Misyail Abdul Rashid | annamisyail@upm.edu.my | Universiti Putra Malaysia | | 7. | Abdul Hanif Khan Yusof Khan | ahanifkhan@upm.edu.my | Universiti Putra Malaysia | | 8. | Wei Chao Loh | lohweichao@upm.edu.my | Universiti Putra Malaysia | | 9. | Janudin Baharin | janudin@upm.edu.my | Universiti Putra Malaysia | | 10. | Aaron Fernandez | aaron@upm.edu.my | Universiti Putra Malaysia | | 11. | Intan Nureslyna Samsudin | intanlyna@upm.edu.my | Universiti Putra Malaysia | | 12. | Mohd Hazmi Mohamed | mdhazmi@upm.edu.my | Universiti Putra Malaysia | | 13. | Mooi Ching Siew | sm_ching@upm.edu.my | Universiti Putra Malaysia | | 14. | Kai Wei Lee | lee_kai_wei@yahoo.com | Universiti Tunku Abdul Rahman | | 15. | Vasudevan Ramachandran | vasuphd@gmail.com | Bharath Institute of Higher Education and Research | | 16. | Patricia Pozo-Rosich | ppozo@vhebron.net | Headache and Craniofacial Pain Unit, Neurology Department, Hospital Universitari Vall d’Hebron, Barcelona, Spain | | 17. | Wan Aliaa Wan Sulaiman | wanaliaa@upm.edu.my | Universiti Putra Malaysia | |
| Contributions | 3b | *Corresponding author mail address: Department of Neurology, Faculty of Medicine and Health Sciences, Universiti Putra Malaysia, 43300 Serdang, Selangor, Malaysia.  Describe contributions of protocol authors and identify the guarantor of the review  **Conceptualization:** Nabil Izzaatie Mohamad Safiai, Wan Aliaa Wan Sulaiman, Mohd Hazmi Mohamed, Liyana Najwa Inche Mat  **Data curation:** Nabil Izzaatie Mohamad Safiai, Nur Afiqah Mohamad, Kai Wei Lee  **Formal analysis:** Nabil Izzaatie Mohamad Safiai, Mooi Ching Siew, Kai Wei Lee, Abdul Hanif Khan, Yusof Khan, Janudin Baharin, Anna Misyail Abdul Rashid, Fan Kee Hoo  **Funding acquisition:** Wan Aliaa Wan Sulaiman  **Methodology:** Nabil Izzaatie Mohamad Safiai, Wan Aliaa Wan Sulaiman, Nur Afiqah Mohamad, Patricia Pozo-Rosich, Vasudevan Ramachandran  **Project administration:** Mooi Ching Siew, Fan Kee Hoo, Liyana Najwa Inche Mat, Mohd Hazmi Mohamed, Intan Nureslyna Samsudin, Aaron Fernandez, Hamidon Basri  **Supervision:** Wan Aliaa Wan Sulaiman, Intan Nureslyna Samsudin, Aaron Fernandez, Hamidon Basri  **Writing – original draft preparation:** Nabil Izzaatie Mohamad Safiai, Nur Afiqah Mohamad  **Writing – review & editing:** Nabil Izzaatie Mohamad Safiai, Nur Afiqah Mohamad and Vasudevan Ramachandran  **Guarantor of review:** Wan Aliaa Wan Sulaiman |
| Amendments | 4 | If the protocol represents an amendment of a previously completed or published protocol, identify as such and list changes; otherwise, state plan for documenting important protocol amendments  No Amendment |
| Support: |  |  |
| Sources | 5a | Indicate sources of financial or other support for the review  Research Grant: Grant Number GPB/2017/9585500 |
| Sponsor | 5b | Provide name for the review funder and/or sponsor  Director of Research Management Centre of Universiti Putra Malaysia Prof. Dr. Mohd Adzir Bin Mahdi  03-9769 1610  [dir.rmc@upm.edu.my](mailto:amirahnaziera@upm.edu.my) / [mam@upm.edu.my](mailto:mam@upm.edu.my) |
| Role of sponsor or funder | 5c | Describe roles of funder(s), sponsor(s), and/or institution(s), if any, in developing the protocol  The funders had and will not have a role in study design, data collection and analysis, decision to publish, or preparation of the manuscript. |
| INTRODUCTION | | |
| Rationale | 6 | Describe the rationale for the review in the context of what is already known  Several systematic reviews had evaluated the use of TMS in headache and migraine, but none reported the use of hf-rTMS applied at DLPFC in migraine prophylaxis. Therefore, we propose this review protocol to investigate the evidence of the efficacy of the treatment for migraine prophylaxis. |
| Objectives | 7 | Provide an explicit statement of the question(s) the review will address with reference to participants, interventions, comparators, and outcomes (PICO)  The population is participants diagnosed with migraine headache.  The intervention is hf-rTMS at the DLPFC area, and the comparator is sham stimulation.  The main outcome is the treatment efficacy (measured by headache days). The secondary outcomes are tolerability (measured by discontinuation rate) and safety (measured by adverse events and side effects). |
| METHODS | | |
| Eligibility criteria | 8 | Specify the study characteristics (such as PICO, study design, setting, time frame) and report characteristics (such as years considered, language, publication status) to be used as criteria for eligibility for the review  This systematic review will include only randomised controlled clinical trials (RCTs) that study migraine treatment using hf-rTMS applied over the DLPFC area in migraine patients. Only articles written in English from inception until December 2020 will be included in this review.  Only full-text articles written in English will be included. Conference and proceedings article will be excluded from this review. Studies primarily examining other comorbid conditions with migraine will also be excluded. |
| Information sources | 9 | Describe all intended information sources (such as electronic databases, contact with study authors, trial registers or other grey literature sources) with planned dates of coverage   1. Electronic data sources: Scopus, Cumulative Index to Nursing and Allied Health Literature Plus, PubMed, Cochrane Central Register of Controlled Trials and Biomed Central. 2. clinicaltrial.gov and the World Health Organization trial registry. 3. citation searching in which the papers that have cited the included articles will be scanned. 4. reference list of the included studies and other relevant papers to conduct a thorough search for this systematic review.   Planned date of coverage: From inception until December 2020. |
| Search strategy | 10 | Present draft of search strategy to be used for at least one electronic database, including planned limits, such that it could be repeated   | No. | Search terms | | --- | --- | | 1. | rTMS or “repetitive transcranial magnetic stimulation” AND migrain* | | 2. | rTMS or “repetitive transcranial magnetic stimulation” AND headache* | | 3. | rTMS or “repetitive transcranial magnetic stimulation” AND hemicran* | | 4. | rTMS or “repetitive transcranial magnetic stimulation” AND migraine disorders | |
| Study records: |  |  |
| Data management | 11a | Describe the mechanism(s) that will be used to manage records and data throughout the review  All searches result will be exported to Endnote referencing software, and duplicates will be removed manually.  Data will be saved in an excel sheet. Data synthesis will be performed using Cochrane Collaboration’s software program Review Manager (RevMan) V.5.4.1 for desktop. |
| Selection process | 11b | State the process that will be used for selecting studies (such as two independent reviewers) through each phase of the review (that is, screening, eligibility and inclusion in meta-analysis)  The study screening and selection process will be performed by two independent reviewers. We will do the initial screening using titles and abstracts screening, and those match the interest and relevant to our systematic review will be included. |
| Data collection process | 11c | Describe planned method of extracting data from reports (such as piloting forms, done independently, in duplicate), any processes for obtaining and confirming data from investigators  The process of data extraction will be performed by two independent reviewers. A pre-prepared excel datasheet will be used by the reviewers. Insufficient data will be requested from the trialist whenever possible. |
| Data items | 12 | List and define all variables for which data will be sought (such as PICO items, funding sources), any pre-planned data assumptions and simplifications  For each study, the following information will be extracted:  Authors’ name  Publication year  Type of migraine  Preventive treatment  Group allocation of treatment  Number of patients randomised (total and per group)  Gender and mean age of participant  Stimulation protocol  Primary outcome and additional outcome  Side effects  Dropout (and reasons) |
| Outcomes and prioritization | 13 | List and define all outcomes for which data will be sought, including prioritization of main and additional outcomes, with rationale  The main outcome is the treatment efficacy (measured by headache days). The secondary outcomes are tolerability (measured by discontinuation rate) and safety (measured by adverse events and side effects). |
| Risk of bias in individual studies | 14 | Describe anticipated methods for assessing risk of bias of individual studies, including whether this will be done at the outcome or study level, or both; state how this information will be used in data synthesis  For quality assessment, 3-4 independent reviewers will assess the articles using the version 2 Cochrane risk-of-bias tool for randomised trials (RoB 2) from the Cochrane Handbook for Systematic Reviews of Interventions Version 6.1. The bias that will be assessed includes bias arising from the randomisation process, bias due to deviations from intended interventions, bias due to missing outcome data, the bias in the measurement of the outcome and bias in the selection of the reported result. Disagreements will be resolved by discussion between the reviewers. |
| Data synthesis | 15a | Describe criteria under which study data will be quantitatively synthesised  Data from the intervention will be compared with the data from the comparator sham group. If feasible, a meta-analysis will be performed to determine the most efficacious and tolerable hf-rTMS protocol. |
|  | 15b | If data are appropriate for quantitative synthesis, describe planned summary measures, methods of handling data and methods of combining data from studies, including any planned exploration of consistency (such as I^2^, Kendall’s τ)  For dichotomous data, the outcome will be presented as relative risks (RRs) with 95% CIs. For continuous data, the effect size of the interventions will be calculated using the mean differences (MDs) with 95% CIs. If the study trials present the outcome values using different scales, the standard mean difference (SMD) with 95% CIs will be used. Meanwhile, the data for the meta-analysis will be calculated using fixed or random effects. |
|  | 15c | Describe any proposed additional analyses (such as sensitivity or subgroup analyses, meta-regression)  For heterogeneity assessment, the degree of heterogeneity between the studies will be calculated using the I² statistic. Value >50% will be considered indicative of substantial heterogeneity. If the level of heterogeneity is high, subgroup analysis will be performed to explore the possible causes of heterogeneity. Subgroup analyses will be performed according to factors affecting the outcomes. |
|  | 15d | If quantitative synthesis is not appropriate, describe the type of summary planned  If quantitative data synthesis is not possible, a narrative analysis will be performed. |
| Meta-bias(es) | 16 | Specify any planned assessment of meta-bias(es) (such as publication bias across studies, selective reporting within studies)  The bias that will be assessed includes bias arising from the randomisation process, bias due to deviations from intended interventions, bias due to missing outcome data, the bias in the measurement of the outcome and bias in the selection of the reported result. |
| Confidence in cumulative evidence | 17 | Describe how the strength of the body of evidence will be assessed (such as GRADE)  The strength of the body of evidence will be assessed using GRADE. |

*** It is strongly recommended that this checklist be read in conjunction with the PRISMA-P Explanation and Elaboration (cite when available) for important clarification on the items. Amendments to a review protocol should be tracked and dated. The copyright for PRISMA-P (including checklist) is held by the PRISMA-P Group and is distributed under a Creative Commons Attribution Licence 4.0.**

*From: Shamseer L, Moher D, Clarke M, Ghersi D, Liberati A, Petticrew M, Shekelle P, Stewart L, PRISMA-P Group. Preferred reporting items for systematic review and meta-analysis protocols (PRISMA-P) 2015: elaboration and explanation. BMJ. 2015 Jan 2;349(jan02 1):g7647.*
